# Comparison of several Real-Time PCR Kits versus a Culture-dependent Algorithm to Identify Enteropathogens in Stool Samples

**DOI:** 10.1038/s41598-020-61202-z

**Published:** 2020-03-09

**Authors:** Silvia Valledor, Inés Valledor, María Concepción Gil-Rodríguez, Cristina Seral, Javier Castillo

**Affiliations:** 1 Facultad de Ciencias, Universidad de Zaragoza, Zaragoza, Spain; 2 CerTest Biotec S.L., San Mateo de Gállego, Zaragoza, Spain; 3 Facultad de Medicina, Universidad de Zaragoza, Zaragoza, Spain; 4 Hospital Clínico Universitario Lozano Blesa, Servicio de Microbiología, Zaragoza, Spain; 5 Instituto de Investigación Sanitaria Aragón (IIS Aragón), Zaragoza, Spain

**Keywords:** Microbiology techniques, Molecular biology

## Abstract

This study aims to validate the current diagnostic method for the clinical detection of gastroenteritis. We analyzed 400 stool samples to detect three of the most common enteropathogens: *Salmonella* spp., *Campylobacter* spp., and *Yersinia enterocolitica*. All specimens were tested with a routine clinical diagnosis algorithm and with five real-time PCR assays. A total of 98 specimens (24.5%) were positive for enteropathogens. We found 24 samples positive for *Salmonella enterica*, 71 positive for *Campylobacter* spp., and 4 positive for *Yersinia enterocolitica*. All evaluated methods exhibited a good performance in identifying *Salmonella* and *Yersinia enterocolitica*, being the highest positive percent agreement (PPA) value of 95.8% and 100%, respectively. The clinical algorithm showed the highest PPA value identifying *Salmonella*, due to the enrichment in selenite broth. However, the evaluated methods showed notable differences in the identification of *Campylobacter* species, obtaining a wide range of PPA values: 59.2%–100%. The clinical algorithm showed the lowest PPA value since it was only able to detect *Campylobacter jejuni* and *Campylobacter coli* species. This study revealed the importance of implementing the real-time PCR technique in a clinical algorithm: it improved the accuracy of the diagnosis and provided results in a shorter time compared to routine clinical methods.

## Introduction

Acute gastroenteritis is a leading cause of morbidity and mortality worldwide^1^, particularly in at-risk populations, such as children, older individuals, and immunocompromised patients. We included three bacteria in this study: *Salmonella* spp., *Campylobacter* spp., and *Yersinia enterocolitica*, which are among the most common enteropathogens that cause gastrointestinal infections. Thus, their rapid, accurate identification is crucial for infection control, and in selected cases, for determining the most suitable therapy. According to the Centers for Disease Control and Prevention, during 2016, the Foodborne Active Surveillance Network identified 24,029 cases of foodborne infections, which led to 5,512 hospitalizations (22.9%) and 98 deaths (0.4%). Some of these cases were detected by culture, but others were detected with culture-independent diagnostic tests (CIDTs). Current CIDTs mainly comprise two types: antigen-based and nucleic acid-based assays. In 2016, one study showed that many infections were identified with a CIDT and without a culture confirmation; most frequently, *Campylobacter* (32%) and *Yersinia* (32%); but also Shiga toxin-producing *E.coli* (STEC) (24%), *Shigella* (23%), *Vibrio* (13%), and *Salmonella* (8%)^2^. That study revealed the consequences of the current change in diagnostic trends. The implementation of CIDTs in clinical laboratories has led to more exhaustive diagnoses. Most of these tests are ordered by clinicians, because CIDTs are easier to perform and they return results faster than traditional culture techniques. Moreover, samples are subjected to deep screening when multiplex panels are used; consequently, the number of reported infections has increased^2,3^. Routine detection of enteropathogens in clinical laboratories typically consists of a complex algorithm, based on selective culture-, biochemistry-, and immunology-based tests. Most studies have shown that culture-based methods lacked sensitivity, particularly in identifying *Campylobacter*, and most researchers agree that nucleic acid-based CIDTs have great potential. Consequently, CIDTs (particularly gastrointestinal panels, based on multiplex real-time PCR technology (qPCR)) are increasingly implemented in clinical laboratories^4–8^, due to their high sensitivity and their ability to screen a considerable number of enteropathogens simultaneously. This approach avoids complex algorithms and reduces the hands-on time. However, the use of stool antigen-based CIDTs remains controversial, because they have displayed highly variable sensitivities and specificities, depending on the study^9,10^.

This study aimed to compare five molecular CIDTs, based on qPCR technology, to culture-dependent techniques to evaluate their performance and determine the most suitable alternative for routine clinical diagnoses of enteropathogens.

## Materials and Methods

### Study population and clinical stool specimens

Stool samples were submitted from October to December 2015 to the Hospital Clinico Universitario Lozano Blesa (Zaragoza, Spain). This hospital had 803 beds and attended a population of 286,774 inhabitants, with 29,506 admissions annually. It included an outpatient care facility with 2,315,197 visits annually and an emergency department with 127,694 visits annually. The samples were randomly chosen from specimens received with a request for routine detection of enteropathogens (bacteria, virus, and parasites). This study was approved by the Clinical Research Ethics Committee of Aragon (25 Sept 2019; PI19/378). This evaluation does not require informed consent as it was performed with medical waste. After routine analysis, the leftover of DNA, which is considered medical waste, was analyzed with molecular methods. These non-hospital routine diagnosis methods implied no risk or burden for any individuals and the patient did not have to provide any additional sample. The study focused on the presence/absence of bacterial DNA, without attending to residual human DNA. For each patient, the following data were collected: gender, age, hospitalization status, and stool sample consistency. The non-clinicians researchers never had access to personal identifiable information or clinical record. All recorded data were robustly pseudonymized at source, so no subject-identifiable data were generated.

### Study design and case definition

Several comparisons were performed simultaneously. All specimens were tested with five qPCR assays. In addition, all specimens were analyzed with the routine clinical algorithm. Discrepant samples among the qPCR assays were resolved with additional qPCR tests. Discrepancies between the qPCR assays and the routine clinical algorithm were resolved with conventional PCR and sequencing.

When an incongruence could not be resolved, the information derived from the other tests was used to resolve it. The growth in culture was considered a positive result, regardless of other results.

Consequently, taking into account the above mentioned factors, we designated the following as “cases”:

- Samples that were identified as positive in culture media.
- Discrepant samples, with positive results supported by resolution tests. We consider as resolution tests those that help us to resolve the incongruences. In this study they were the additional qPCR test and the conventional PCR and sequencing.
- Discrepant samples, without successful resolution tests, but a positive result was supported by at least three qPCR assays.

### Routine clinical algorithm

Once samples arrived at the Microbiology laboratory, they were routinely plated onto various selective and differential media, including selenite broth, MacConkey (MCK) agar, Hektoen enteric (HEK) agar, cefsulodin-irgasan-novobiocin (CIN) agar, xylose lysine deoxycholate (XLD) agar, and charcoal cefoperazone deoxycholate agar (CCDA). These cultures were grown to isolate and identify the enteric pathogens. All culture media were obtained from Oxoid (Thermo Fisher Scientific, Massachusetts, US).

Stool samples were inoculated into enrichment selenite broth and incubated at 37 °C for 24 h prior to culturing in XLD agar. All media were incubated at 37 °C for 24 h, except for CCDA plates, which were incubated at 42 °C for 48–72 h under microaerophilic conditions, with a GasPak EZ Campy system (BD Diagnostics, New Jersey, US).

*Salmonella* is expected to grow on MCK, HEK and XLD agar, *Campylobacter* on CCDA plates and *Yersinia enterocolitica* on MCK and CIN agar.

Colonies recognized as presumptive pathogens were confirmed with matrix-assisted laser desorption/ionization-time of flight mass spectrometry (MALDI-TOF MS Biotyper 3.1; Bruker, Massachusetts, US)^11^ and they were further analyzed with an automated system (MicroScan®Neg Combo Type 53 Panel; Beckman Coulter, California, US) to determine the sensitivity to antibiotics. Results from the MicroScan® panels were read on the MicroScan Walk Away 96 plus platform (Siemens, Munich, Germany).

The species of *Campylobacter* were determined using MALDI-TOF^11^.

The serotype of *Salmonella* was determined following the Kauffmann-White scheme with the Difco Salmonella O Antiserum Group Kit (Becton Dickinson, New Jersey, US).

The serotype of *Yersinia enterocolitica* was determined with the Yersinia enterocolitica O:3 Antiserum Kit (BioRad, California, US).

### Nucleic acid extraction

Nucleic acids were extracted from fresh stool samples with the VIASURE RNA-DNA Extraction Kit (CerTest Biotec S.L., Zaragoza, Spain). Solid (0.1 g) or liquid (200 µl) stool was added to 200 µl of phosphate-buffered saline, and extraction was performed according to the manufacturer’s instructions. Samples were eluted in 100 µl and stored at −20 °C until use in the PCR detection assay.

### Real-Time PCR assays

Five different Real-Time PCR assays were evaluated in this study: VIASURE *Salmonella* Real-Time PCR Detection Kit, VIASURE *Campylobacter* Real-Time PCR Detection Kit, VIASURE *Yersinia enterocolitica* Real-Time PCR Detection Kit, VIASURE *Salmonella, Campylobacter & Y.enterocolitica* Real-Time PCR Detection Kit (CerTest Biotec S.L., Zaragoza, Spain), and RIDA®GENE Bacterial Stool Panel (R-Biopharm, Darmstadt, Germany).

The targets of the three monoplex and one multiplex VIASURE assays are the *invA* gene (*Salmonella*), 16S rRNA gene (*Campylobacter)*, and *ail* gene (*Yersinia enterocolitica)*, whereas RIDA®GENE Bacterial Stool Panel allows the simultaneous detection of *ttr* gene *(Salmonella)*, 16S rRNA gene *(Campylobacter)*, and *ail* gene (*Yersinia enterocolitica)*. All Real-Time PCR assays were performed following the manufacturer’s protocol. Positive and negative controls were included in each run. All runs were performed on the AriaMx thermocycler (Agilent Technologies, California, US).

### Real-Time PCR resolution test

The Mericon *Campylobacter* spp kit (Qiagen, Hilden, Germany) was used for resolving *Campylobacter* discrepant samples. This Real-Time PCR assay was performed according to the manufacturer’s protocol.

### Conventional PCR

Conventional PCR was performed with the VIASURE ESSENTIALS DNA Master Mix kit (CerTest Biotec S.L., Zaragoza, Spain), according to the manufacturer’s instructions. The kit contained all necessary PCR reagents lyophilized in the wells, except for the primers, which were customizable, depending on the target. The primers used for detecting *Salmonella* (*hilA* gene) and *Campylobacter* (16S rRNA gene) were described previously^12,13^; we used 1 µM of each primer. We modified *Salmonella* primers by deleting the first nucleotides; the following forward (5′-ATTTGCGCCATGCTGAGGTAG-3′) and reverse (5′- CCGCCGGCGAGATTGTG-3′) primers were used.

PCR products were separated on a 1.0% agarose gel by electrophoresis.

### Sequencing

The same conventional PCR primers were used for sequencing. Sequencing was performed by the General Research Support Service at the Health Research Institute of Aragon (IIS Aragon) and the University of Zaragoza. Sequencing was performed with the capillary sequencer, 3500 XL (Applied Biosystems, California, US). Sequences were analyzed with Chromas software, and they were identified with the basic local alignment search tool (BLAST; http://www.ncbi.nlm.nih.gov/BLAST/).

### Statistical analysis

We compared the tests under study in terms of the positive percent agreement (PPA) and negative percent agreement (NPA), instead of sensitivity and specificity. This approach conforms to FDA recommendations^14^, which suggest that sensitivity and specificity should only be used when the comparator method is a reference standard. In this study, we did not use the culture as the reference method. Instead, we used the “case” definition for the comparator method. Samples were designated “cases” when they were positive in the culture media, in the discrepant sample analysis, or at least in three qPCR assays.

The quantification cycle (C_q_) value determines the initial copy number of the target. Samples with low target concentrations display high C_q_ values. A C_q_ value greater than 40 was considered negative.

All statistical analyses were performed with SPSS software (version 22; IBM Corp., Madrid, Spain), except for the 95% confidence intervals (CI), which were calculated with the modified Wald method, with GraphPad QuickCalcs. The Chi-square test was performed to determine correlations between enteropathogen infections and patient groups. A *P*-value <0.05 was considered statistically significant.

## Results

A total of 400 stool samples, acquired from 385 patients, were included in this study. Samples were grouped by stool consistency, and they were also classified according to the patient’s age group, gender, and hospitalization status. These data are shown in Table 1.

**Table 1:** Characteristics of patients and stool specimens.

Age group	No. samples	Mean age ± SD
Infants (<2 years)	62	0.9 ± 0.2
Pre-schoolers (≥2 to <5 years)	59	2.7 ± 0.8
School-age children and adolescents (≥5 to <18 years)	78	9.3 ± 3.1
Adults (≥18 years)	201	55.8 ± 20.6
**Total**	**400**	
**Gender**	**No. samples**	**Percentage (%)**
Female	197	49.3
Male	203	50.8
**Total**	**400**	**100.0**
**Hospitalization status**	**No. samples**	**Percentage (%)**
Hospitalized	25	6.3
Outpatient	331	82.8
Emergency department	44	11.0
**Total**	**400**	**100.0**
**Stool sample consistency**	**No. samples**	**Percentage (%)**
Soft	143	35.8
Ordinary	110	27.5
Tough	40	10.0
Liquid	34	8.5
Crumbly	28	7.0
Mucous	16	4.0
With traces of blood	7	1.8
Missing values	22	5.5
**Total**	**400**	**100.0**

Abbreviations: No, number; SD, standard deviation.

Among 400 stool samples, 98 (24.5%) met the case definition. Of these, 69 were found positive in culture media, 25 were discrepant samples that were resolved, and 5 were discrepant samples that were found positive at least in three qPCR assays. Although there were 98 cases, there were 99 positive results, because one sample corresponded to a co-infection by *Campylobacter* and *Yersinia enterocolitica*. A more detailed analysis is given in Table 2.

**Table 2:** Comparison of different molecular assays and the routine clinical method for enteropathogen identification.

	No. Cases	Detection with different assays				
		VIASURE monoplex	VIASURE multiplex	R-Biopharm multiplex	Conventional diagnostic^b^	Resolution test
***Salmonella***						
9 *S*. Typhimurium, 5 *S*. Enteritidis, 2 *S*. serogroup G, 1 *S*. Mbandaka, 1 *S*. Braenderup, 1 *S*. Paratyphi A	**19**	+	+	+	+	N/A^c^
1 *Salmonella* spp	**1**	+	+	+	-	Sequencing^d^
1 *S*. serogroup G	**1**	+	+	-	+	N/A^c^
1 *S*. Typhimurium	**1**	-	-	+	+	N/A^c^
1 *S*. Typhimurium, 1 *S*. Enteritidis	**2**	-	-	-	+	N/A^c^
**Total**	**24**	**21**	**21**	**21**	**23**	
***Campylobacter***						
35 *C. jejuni*, 3 *C. coli*, 3 *Campylobacter* spp	**41**	+	+	+	+	N/A^c^
5 *C. concisus*, 3 *C. jejuni*, 2 *C. coli*, 1 *C. curvus*, 1 *C. gracilis*, 4 *Campylobacter* spp	**16**	+	+	+	-	Sequencing^e^
1 *C. jejuni*	**1**	+	+	-	+	N/A^c^
4 *C. upsaliensis*, 2 *C. hyointestinalis*, 1 *C. coli*, 1 *C. concisus*, 1 *C. helveticus*, 1 *Campylobacter* spp	**10**	+	+	-	-	Mericon assay/Sequencing^f^
1 *Campylobacter* spp	**1**	+	-	+	-	Mericon^g^
1 *C. concisus*, 1 *C. rectus*	**2**	+	-	-	-	Mericon/ Sequencing^h^
**Total**	**71**	**71**	**68**	**58**	**42**	
***Yersinia enterocolitica***						
4 *Yersinia enterocolitica* O:3	**4**	+	+	+	+	N/A^c^
**Total**	**4**	**4**	**4**	**4**	**4**	

Abbreviations: +, positive detection; −, negative detection; *S*., *Salmonella*; *C*., *Campylobacter*; N/A, not applicable.

^b^Conventional diagnostic assays included the culture method, MALDI-TOF mass spectrometry, agglutination assays, and biochemical tests.

^c^A discrepancy analysis was not applicable, because these samples were positive in culture, which immediately defined them as a “case”.

^d^Conventional PCR was unsuccessful; therefore, the *Salmonella* serotype was not resolved in this sample; however, it was considered a “case”, based on three diagnostic methods, which supported a positive result.

^e^Sequencing was not successful in 4/16 samples, but the Mericon assay (the qPCR resolution test) performed with these four samples provided a positive result; thus, all were considered “cases”.

^f^All of these samples were positive in the Mericon assay, and the positive result was confirmed with sequencing in 9/10 samples.

^g^This sample was positive in the Mericon assay.

^h^These two samples were positive in the Mericon assay, and the positive result was confirmed by sequencing in both samples.

We found 24 cases positive for *Salmonella enterica* (11 *S*. Typhimurium, 6 *S*. Enteritidis, 1 *S*. Mbandaka, 1 *S*. Braenderup, 1 *S*. Paratyphi A, 3 *S*. serogroup G, and 1 *Salmonella* spp.); 71 cases positive for *Campylobacter* (39 *C. jejuni*, 7 *C. concisus*, 6 *C. coli*, 4 *C. upsaliensis*, 2 *C. hyointestinalis*, 1 *C. gracilis*, 1 *C. helveticus*, 1 *C. curvus*, 1 *C. rectus*, and 9 *Campylobacter* spp); and 4 cases positive for *Yersinia enterocolitica* O:3. The information of those *Campylobacter* species that were determined by 16 S rRNA sequencing is collected in Table 3.

**Table 3:** *Campylobacter* species identification by 16S rRNA sequencing.

No. Samples	Identification	Identity	GenBank Accession
3	*C.coli*	95–99%	NZ_CP007181.1
7	*C.concisus*	89–99%	CP012541.1
			GU255908.1
			NC_009802.1
			NR_074156.1
1	*C.curvus*	95%	AF550652.1
1	*C.gracilis*	97%	CP012196.1
1	*C.helveticus*	100%	DQ174164.1
2	*C.hyointestinalis*	98–99%	NZ_JHQP01000019.1
3	*C.jejuni*	99%	NC_002163.1
			CP014744.1
1	*C.rectus*	96%	KF030232.1
4	*C.upsaliensis*	98–99%	NR_118528.1

We could detect most *Salmonella* positive samples with all methods. The routine clinical algorithm values were, as follows: PPA = 95.8%, CI = 78.1–99.9 and NPA = 100%, CI = 98.8–100. The VIASURE and R-Biopharm assays provided the same level of results. All gave a PPA = 87.5%, CI = 68.2–96.5 and a NPA = 100%, CI = 98.8–100.

Nine different species of *Campylobacter* were found. All of these were detected with the VIASURE monoplex assay (PPA = 100%, CI = 93.9–100; NPA = 100%, CI = 98.6–100); the next most accurate assays, in descending order were: the VIASURE multiplex (PPA = 95.8%, CI = 87.8–99.0; NPA = 100%, CI = 98.6–100); the R-Biopharm (PPA = 81.7%, CI = 71.0–89.1; NPA = 100%, CI = 98.6–100), and finally, the routine clinical algorithm (PPA = 59.2%, CI = 47.5–69.8; NPA = 100%, CI = 98.6–100).

The four *Yersinia enterocolitica* O:3 positive samples could be detected with all methods studied (PPA = 100%, CI = 45.4–100; NPA = 100%, CI = 98.8–100).

No false positive results were found in this evaluation; consequently, all NPA values were 100%. Moreover, no inhibition occurred in any qPCR reaction; therefore, it was not necessary to re-test any sample.

Samples were grouped according to the C_q_ value in three categories: C_q_ <25 (high copy samples), C_q_ = 25–35 (medium copy samples) and C_q_ >35 (low copy samples, near the detection limit). They were also classified into two groups: positive by culture and negative by culture. The minimum and maximum C_q_ values were calculated for each group. These data are shown in Table 4. We excluded *Yersinia enterocolitica* samples that were negative by culture as none of them were positive by qPCR assays.

**Table 4:** Summary of the C_q_ values obtained by different Real-Time PCR assays.

	VIASURE monoplex | VIASURE multiplex | R-Biopharm multiplex					
	No. Samples			No. Cases	Min C_q_	Max C_q_
	C_q_ < 25	C_q_ = 25–35	C_q_ > 35			
***Salmonella***						
Positive culture	5 | 7 | 4	9 | 11 | 8	6 | 2 | 8	**20** | **20** | **20**	17.3 | 16.3 | 21.1	39.7 | 35.8 | 39.6
Negative culture	0 | 0 | 0	1 | 1 | 1	0 | 0 | 0	**1** | **1** | **1**	30.0 | 28.7 | 31.5	30.0 | 28.7 | 31.5
***Campylobacter***						
Positive culture	29 | 28 | 29	13 | 13 | 11	0 | 1 | 1	**42** | **42** | **41**	15.1 | 14.2 | 15.0	34.9 | 36.9 | 35.8
Negative culture	11 | 11 | 4	15 | 14 | 12	3 | 1 | 1	**29** | **26** | **17**	21.4 | 19.9 | 21.2	38.1 | 36.3 | 36.1
***Yersinia enterocolitica***						
Positive culture	2 | 2 | 2	2 | 2 | 1	0 | 0 | 1	**4** | **4** | **4**	21.9 | 21.3 | 24.0	33.0 | 33.3 | 35.8

Abbreviations: C_q_, quantification cycle; No, number; Min, minimum; Max, maximum.

We also evaluated whether some factors (age, hospitalization status, and stool sample consistency) might be positively associated with infections by these three enteropathogens. We performed a Chi-square test, but we did not find any statistically significant link between enteropathogen infections and the age group (*P* = 0.199), hospitalization status (*P* = 0.556), or stool sample consistency (*P* = 0.073). Hence, we can only report trends. When analyzed according to age, most patients affected seem to be the youngest; infection rates were 32.2% in pre-school children; 30.6% in infants; 23.1% in school-age children and adolescents; and 20.9% in adults. When analyzed according to hospitalization status, infection rates were higher among patients that required hospitalization (28.0%), compared to those attended in outpatient clinics (25.1%) and in the emergency department (18.2%). Finally, when analyzed according to sample consistency, infections were found most frequently in stools with mucous (43.8%), liquid (38.2%), and crumbly (35.7%) consistencies and in stools with traces of blood (28.6%).

## Discussion

This study aimed to validate the current routine clinical diagnosis method for identifying the three main bacterial enteropathogens: *Salmonella*, *Campylobacter*, and *Yersinia enterocolitica*. We compared the standard culture-based techniques to three types of qPCR assays (VIASURE monoplex, VIASURE multiplex, and R-Biopharm).

In recent years, a large number of studies have been conducted to validate novel gastrointestinal molecular panels as BD Max enteric bacterial panel^4^, ProGastro SSCS assay^7^, Allplex GI-Bacteria assay^8^ or FilmArray GI Panel^5,15^. These previous studies emphasized the importance of not considering the culture technique as the reference method, due to its lack of sensitivity. Consequently, we created a reference method based on different techniques. According to this reference, samples were classified as “cases” or “non-cases” (see Materials and Methods section).

All methods showed acceptable PPA values (87.5%–95.8%) for identifying *Salmonella* spp. The highest PPA value was obtained with the routine clinical algorithm. This could be explained by the fact that stool samples were inoculated into a selenite enrichment broth, as reported in previous studies^16^. This broth contributed to bacterial replication, which enhanced the culture method. The culture method was able to detect samples that showed C_q_ values extremely near the detection limit (C_q_ > 35).The maximum C_q_ values obtained in *Salmonella* identification were 39.7, 35.8, and 39.6 with VIASURE monoplex, VIASURE multiplex, and R-Biopharm assays, respectively (Table 4). This observation revealed the high sensitivity that could be achieved with the culture method, due to the enrichment in selenite broth. We performed qPCR assays to identify *Salmonella* in two selenite broths whose stool sample results were negative with qPCR. As we suspected, the *Salmonella* DNA could be detected in the selenite broth with qPCR assays but could not be detected in the stool sample. The benefits of the Real-Time PCR in combination with the enrichment in selenite broth have been recently reported. Boer *et al*.^17^ found that the number of *Salmonella* positive samples increased by 2.2% when qPCR was performed after enrichment in selenite broth. Tang *et al*.^18^ have also validated the high sensitivity and specificity of a national standard protocol based on Real-Time PCR combined with guided culture.

Relative to *Campylobacter*, the qPCR assays exhibited notable differences in their abilities to detect many *Campylobacter* species. The highlight was the high capacities of the VIASURE monoplex and multiplex assays (PPA = 100% and PPA = 95.8%, respectively) in diagnosing the majority of *Campylobacter* species that are considered pathogenic in humans^19^.

The R-Biopharm test displayed less diagnostic capacity (PPA = 81.7%). It seems to be due to a failure in the recognition of less common *Campylobacter* species (other than *C.jejuni* and *C.coli*) rather than in the sensitivity of the test, since most of the samples were in high concentration (C_q_ < 25). The R-Biopharm assay could not detect any positive sample for *C. upsaliensis*, *C. hyointestinalis*, *C. helveticus*, or *C. rectus* (Table 2).

Only 3/13 samples that R-Biopharm missed were near the detection limit (C_q_ > 35) (1 *C. concisus*, 1 *C. rectus* and 1 *Campylobacter* spp.); 3/13 showed a C_q_ = 25–35 (1 *C. concisus*, 1 *C. helveticus* and 1 *C. jejuni*) and 7/13 samples showed a C_q_ < 25 (4 *C. upsaliensis*, 2 *C. hyointestinalis* and 1 *C. coli*) (Tables 2, 4).

The routine clinical algorithm was only capable of detecting *C. jejuni* and *C. coli* species (PPA = 59.2%) (Table 2). This finding was consistent with the literature, where different studies^20–22^ indicated that some antimicrobial agents incorporated into the commonly used selective media (like cefoperazone in CCDA media) could inhibit the growth of several species, including *C. hyointestinalis*, *C. upsaliensis*, and *C. fetus*. Moreover, not all *Campylobacter* species are thermophilic; consequently, the incubation at 42 °C was not suitable for all species. Additionally, some species, like *C. concisus, C. rectus, C. curvus, C. gracilis*, and *C. showae* require a special atmosphere enriched with hydrogen.

The culture technique failed to detect 3 *C. jejuni* and 3 *C. coli* samples (Table 2). None of them were low copy samples since 4/6 showed values of C_q_ < 25 and 2/6 showed a C_q_ value = 25–35. This observation revealed that sometimes even the growth of *C. jejuni* and *C. coli* in selective media could be complicated.

For the above reasons, the qPCR technique appears to be a reliable solution for *Campylobacter* identification since it could ensure the identification of most *Campylobacter* species without excessive complications in the diagnostic algorithm.

Regarding *Yersinia enterocolitica*, all the methods studied could detect all samples positive for *Yersinia enterocolitica* O:3 (PPA = 100%).

An important aspect of this study was that no false positive result was found in any qPCR assay. This high specificity (100%) could represent a key difference between molecular-based and antigen-based CIDTs. Couturier^10^ stated that the problem with stool antigen-based CIDTs was that they exhibited highly variable sensitivities and specificities. That problem has not occurred with molecular-based CIDTs, which, to date, have shown excellent sensitivity and specificity compared to cultures. Our findings correlate with those previous results; therefore, molecular CIDTs could be considered a reliable diagnostic tool, although more studies are needed. Additionally, some molecular CIDTs, like VIASURE assays, are very easy to use. All the components are provided lyophilized inside the wells, which implies that the user is not required to mix any reagents. Also, the lyophilized format facilitates the storage and shipping conditions as lyophilized reagents are stable at room temperature.

We did not encounter inhibition in any of the qPCR assays, although we tested a vast range of stool consistencies. This finding suggested that the qPCR assays used in this study are robust tests with a stable matrix formulation, which neutralized PCR inhibitor effects.

The main limitation of this study was that the number of positive samples depended on the prevalence of each pathogen in the study area. Consequently, we recommend that a multicenter study should be performed, and that the sample collection time should be extended.

We conclude that the three types of qPCR assays (VIASURE monoplex, VIASURE multiplex and R-Biopharm) tested could improve the routine clinical diagnosis algorithm, because they had a noticeable impact on the *Campylobacter* diagnosis. Additionally, they exhibited good performance in *Salmonella* and *Yersinia enterocolitica* identification too, and they substantially reduced the turnaround time (from days to hours). The ease of using molecular methods and the time they save will allow laboratories to increase the number of samples processed. Moreover, saving time is critical for avoiding the spread of infections and for providing effective treatment plans.
